# Polymorphisms in KSHV-encoded microRNA sequences affect levels of mature viral microRNA in Kaposi Sarcoma lesions

**DOI:** 10.18632/oncotarget.26321

**Published:** 2018-11-09

**Authors:** Vickie A. Marshall, Nazzarena Labo, Joanna Sztuba-Solinska, Elena M. Cornejo Castro, Karen Aleman, Kathleen M. Wyvill, Lynne McNamara, Stuart F.J. Le Grice, Robert Yarchoan, Thomas S. Uldrick, Patrick MacPhail, Mark N. Polizzotto, Denise Whitby

**Affiliations:** ^1^ AIDS and Cancer Virus Program, Leidos Biomedical Research, Frederick National Laboratory for Cancer Research, Frederick, MD, USA; ^2^ Basic Research Laboratory, Center for Cancer Research, Frederick National Laboratory for Cancer Research, Frederick, MD, USA; ^3^ HIV and AIDS Malignancy Branch, National Institutes of Health, Bethesda, MD, USA; ^4^ Department of Internal Medicine, Faculty of Health Sciences, University of Witwatersrand, Johannesburg, South Africa; ^5^ Clinical HIV Research Unit, Department of Medicine, University of the Witwatersrand, Johannesburg, South Africa; ^6^ Department of Biological Sciences, Auburn University, Auburn, AL, USA

**Keywords:** KSHV, microRNA, Kaposi's sarcoma

## Abstract

**Background:**

We previously reported Kaposi sarcoma-associated herpesvirus (KSHV) microRNA sequence variants in clinical samples correlated with increased risk of multicentric Castleman's disease (MCD). We then demonstrated that microRNAs with variant sequence have different maturation and mature microRNA expression *in vitro*. Here, we illustrate the association between microRNA sequence and changes in mature microRNA levels within Kaposi sarcoma (KS) lesions.

**Methods:**

KSHV microRNA sequences were determined from 20 KS lesions and 4 control skin biopsies from individuals evaluated for KS. Levels of mature KSHV microRNAs were measured with 21 custom small RNA qRT-PCR assays using RNA RNU6B as endogenous control.

**Results:**

The levels of 13 KSHV-encoded microRNAs were elevated in KS lesions compared to control biopsies. MicroRNA 9-5p was strongly down regulated in South African vs. US biopsies. Low levels of K12-9-5p were associated with single nucleotide polymorphisms (SNPs) in miR-K12-9-5p, 4-5p, 5-3p, 7-3p and pri-miR-K12-3. One SNP in pri-miR-K12-3 resulted in down regulation of miR-K12-6-3p, 8-3p, 10-3p, 12-5p and the upregulation of 5-5p, illustrating sequence variants outside pre-microRNAs were also associated with changes in mature microRNA levels.

**Conclusions:**

The levels of mature KSHV-encoded microRNAs in KS lesions correlate with sequence variation reflecting changes in secondary and tertiary RNA structure.

## INTRODUCTION

Kaposi's sarcoma-associated herpesvirus (KSHV) is the causative agent of two B-cell lymphoproliferative diseases, primary effusion lymphoma (PEL) and a variant of multicentric Castleman's disease (MCD), as well as Kaposi's sarcoma (KS). KS is a highly vascularized tumor of endothelial cell origin classified into four clinico-epidemiological forms of similar pathology. Classic KS mostly occurs in older, HIV negative men of predominantly Mediterranean ancestry and has a typically indolent course. Endemic KS is seen in HIV negative individuals in sub-Saharan Africa and has a more aggressive presentation and course. Iatrogenic KS is associated with immune-suppressive treatment in organ transplant recipients and usually resolves once the regimen is reduced or discontinued. Finally, AIDS-KS is a more aggressive disease associated with considerable mortality even in the context of cART. [1]

Many studies have attempted to explain the differences between African KS cases compared to those occurring in other regions by examining genetic, behavioral and environmental exposures, including co-infections with other pathogens [2–5]. While several possible cofactors have been identified, no one factor alone adequately explains the differences observed in disease severity [6].

KSHV subtypes, identified by the sequence of the K1 gene, which varies up to 30% at the amino acid level, have a characteristic geographical distribution. KSHV subtypes A and C are found in Europe, Asia, and North and Central America while A5/B/F subtypes occur in Africa. Studies examining K1 variations have shown that viral subtypes are closely associated with ethnicity and geography but correlations with disease risk or type are inconsistent [7–9].

The discovery of KSHV-encoded microRNAs offered the opportunity to examine sequence variations in a coding region of KSHV with the potential to affect viral and host gene expression patterns. KSHV encodes 12 pre-microRNAs resulting in the potential production of 25 mature microRNAs. All KSHV microRNAs are located within the latency-associated region that encodes essential proteins for viral persistence. A cluster of 10 microRNAs is encoded in a 2.8 kb intragenic region between the kaposin (K12) and viral FLIP genes (K13), while the remaining two, miR-K12-10 and miR-K12-12, are located within the open reading frame and 3’ UTR of K12 respectively [10–13].

We have previously described sequence variation in the KSHV microRNA genes and shown how these can be functionally relevant. First, we identified microRNA polymorphisms related to specific viral subtypes, in both clinical samples and cell lines [14]. Phylogenetic analysis showed microRNA sequences clustered into an A/C branch, including sequences derived from patients in North America and Europe; and a B/F branch, consisting predominantly of sequences identified in KSHV infecting African individuals and MCD patients [14]. We showed an association with microRNA sequence polymorphisms and AIDS-KS in a European case control study [8] and with increased risk of MCD and the newly described KSHV inflammatory cytokine syndrome (KICS) [15]. In addition, we demonstrated that SNPs in some pre-microRNA sequences affected microRNA processing and mature microRNA production in a complex manner *in vitro* [16].

In the current study, we examined microRNA sequence variation in KS patients in the US and South Africa and assessed mature KSHV microRNAs levels within KS lesions to investigate potential correlations. Furthermore, to explore whether such correlations were associated with the cumulative effects of SNPs on secondary structure, we derived *in silico* the RNA structure of both a representative US and variant South African KSHV microRNA cluster sequence.

## RESULTS

### KSHV subtypes

**Figure 1:**
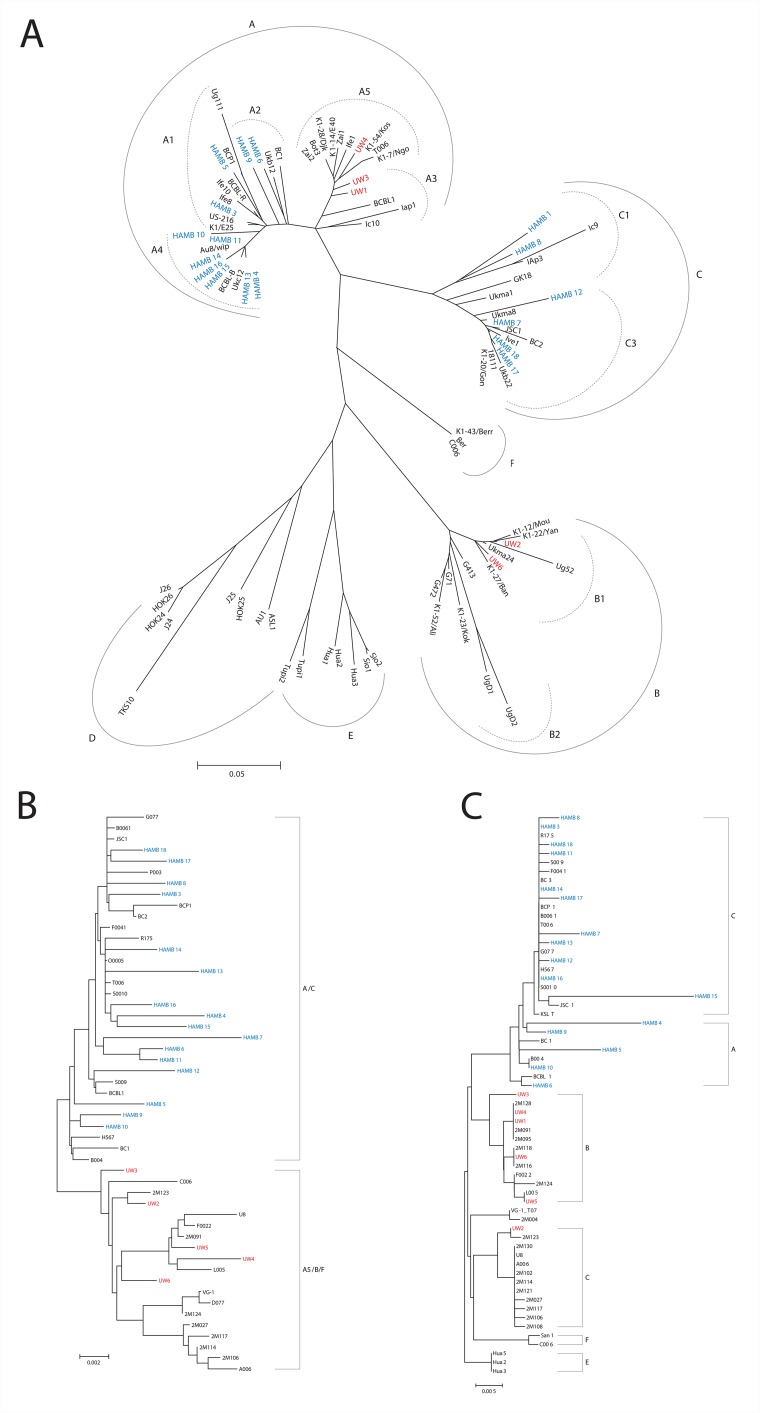
**(A)** K1 Radial Phylogenetic tree. The Neighbor-Joining method was used to infer the evolutionary history. Nucleotide sequences were converted to amino acid prior to analysis. The phylogenetic tree analysis involved 89 amino acid sequences including current study participants and representative references from GenBank. All positions containing gaps and missing data were eliminated. There were a total of 147 positions in the final dataset. Subtypes are as indicated and are consistent with previous reports. Evolutionary analyses were conducted in MEGA6. **(B)** MicroRNA Cluster Phylogenetic tree. The microRNA cluster region phylogenies were determined using bootstrap neighbor-joining analysis with 100 replicates. The analysis involved 52 nucleotide sequences. There were a total of 2495 positions in the final dataset. Evolutionary analyses were conducted in MEGA6. **(C)** T0.7 Neighbor-joining tree: The evolutionary history was inferred using the Neighbor-Joining bootstrap analysis using 100 replicates. The subtype nomenclature is consistent with that established for the KSHV K12/T0.7 gene region. The analysis involved 64 nucleotide sequences from current study samples and GenBank references of appropriate subtypes. The final dataset included 530 positions. Evolutionary analyses were conducted in MEGA6.

US patients, enrolled at the HIV and AIDS Malignancy Branch (HAMB) of the NCI, all had KSHV K1 A or C subtype sequences; while South African patients, from the University of Witwatersrand (UW), had KSHV A5, B, or F subtypes, as summarized in Table 1 and as shown in Figure 1A. K1 subtype was not identified in UW5 despite numerous amplification attempts with multiple K1 primer sets, possibly due to sequence variation. K1 subtype was also not determined for HAMB2 due to low viral copy.

**Table 1:** Patients and Sample Characteristics

Sample	Tissue	Ethnicity	HIV Status	KSHV Copy per 10^6^ Cells in biopsy tissue	K1	MicroRNA Cluster	K12/T0.7
UW1	control	African	pos	545	A5	partial	A5
HAMB1	control	African American	pos	QP	C1	partial	ND
HAMB2	control	Hispanic	pos	undetectable	ND	partial	ND
HAMB3	control	African American	neg	850	A1	A/C	C
UW2	KS	African	pos	16,364,000	B1	A5/B/F	B
UW3	KS	African	pos	230,000	A5	A5/B/F	A5
UW4	KS	African	pos	307,000	A5	A5/B/F	A5
UW5	KS	African	pos	76,000	ND	A5/B/F	A5
UW6	KS	African	pos	109,000	B1	A5/B/F	A5
HAMB4	KS	African American	pos	6,100	A4	A/C	A
HAMB5	KS	Caucasian	neg	533,000	A1	A/C	A
HAMB6	KS	African American	pos	8,500,000	A2	A/C	A
HAMB7	KS	Caucasian	pos	744,000	C3	A/C	C
HAMB8	KS	Caucasian	neg	248,000	C1	A/C	C
HAMB9	KS	Caucasian	pos	19,000	A2	A/C	A
HAMB10	KS	Caucasian	pos	40,000	A4	A/C	A
HAMB11	KS	African American	pos	341,500	A4	A/C	C
HAMB12	KS	Hispanic	pos	12,429,000	C3	A/C	C
HAMB13	KS	Caucasian	neg	584,000	A4	A/C	C
HAMB14	KS	Caucasian	neg	683,500	A4	A/C	C
HAMB15	KS	Caucasian	neg	121,000	A4	A/C	C
HAMB16	KS	Hispanic	pos	515,000	A4	A/C	C
HAMB17	KS	African American	pos	17,000	C3	A/C	C
HAMB18	KS	Caucasian	pos	1,907,000	C3	A/C	C

The characteristics of all patients including ethnicity, HIV status, and estimated KSHV viral load in biopsy tissues are as indicated. The viral K1, microRNA cluster, and K12/T0.7 subtypes are indicated as A-F depending on phylogenetic analysis classification (referencing Figures 1A-1C). KSHV copies per million cells rounded to three significant figures. Partial microRNA sequences were obtained for UW1, HAMB1, and HAMB2 but subtype was not determined due to insufficient overlap of sequence reads. K1 sequence was not successfully determined for HAMB2 and UW5.

Abbreviations: ND=not determined. UW=samples from University of Witwatersrand, South Africa. HAMB=samples from HIV and AIDS Malignancy Branch, NCI-Bethesda, Maryland. KS=Kaposi's sarcoma. Pos=positive; Neg=negative; QP=qualitative positive sample with average estimated copy number less than 3.

### MicroRNA sequence analysis

**Figure 2:**
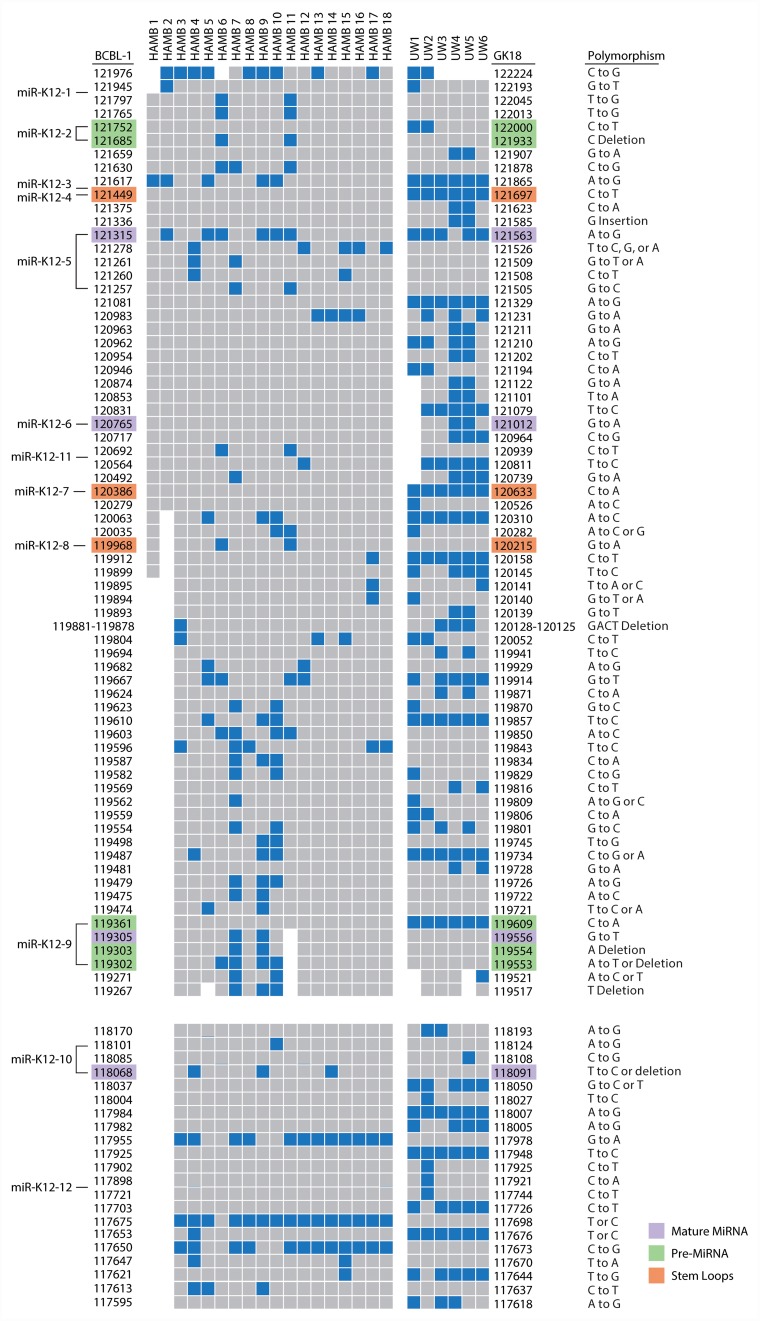
Map of polymorphisms occurring in the microRNA cluster region (top) and the K12/T0.7 gene (bottom) Polymorphisms, as indicated in blue, were defined by sequence comparison to the BCBL-1 cell line as noted on the left of columns; the GK18 cell line is referenced on the right. Specific locations of polymorphisms in relation to reference sequence are noted with nucleotide positions. Areas of missing sequence are shown as gaps. Location of SNPs within microRNA structures are color coded as indicated. Abbreviations: UW=samples from University of Witwatersrand, South Africa. HAMB=samples from the HIV and AIDS Malignancy Branch. BCBL-1 and GK18 GenBank accession number referenced are AY973824.1 and AF148805.2 respectively.

Phylogenetic trees for the microRNA cluster and T0.7 gene are shown in Figures 1B and 1C. Sequences from all patients in the current study born in Africa clustered with previously characterized MCD and KICS patients, several of whom were also from African countries [15]. Only part of the microRNA cluster region was successfully amplified from the UW1 control sample due to low viral copy number and limited material. Similarly, only partial sequences of the microRNA cluster region were obtained from the HAMB1 and HAMB2 controls due to extremely low or non-detectable tumor viral load, and unavailability of corresponding saliva or PBMC samples. The BCBL-1 cell line microRNA sequence (GenBank reference AY973824.1) was arbitrarily defined as wild type (wt), consistent with previous studies [11, 8, 15]. Previously reported KSHV microRNA polymorphisms were observed, particularly within samples from South African patients. These SNPs include changes in pre-microRNA sequences for miR-K12-4-5p, 5-3p, 6-3p, 7-3p, 9-5p, and 10-5p [8, 14, 15]. In the current study, 38 additional polymorphisms have been identified (Figure 2), including one change in pre-microRNAs K12-2 and K12-8 and 3 changes in both K12-5 and K12-9. Novel SNPs were recorded if detected in three or more samples, including sequences available in GenBank, and confirmed in all clones sequenced from each sample.

Consistent with previous observations, the sequence between microRNAs K12-8 and 9 showed the greatest variation between samples. The number of SNPs did not differ significantly between sequences from KS and control samples. Samples from South African patients had a significantly greater number of SNPs than US patients (UW vs HAMB, p<0.0001). Differences by geographical origin were explained by genotype (A5, B, F vs A, C p<0.0001), and no other patient characteristic, including HIV infection, was associated with differences in sequence.

**Figure 3:**
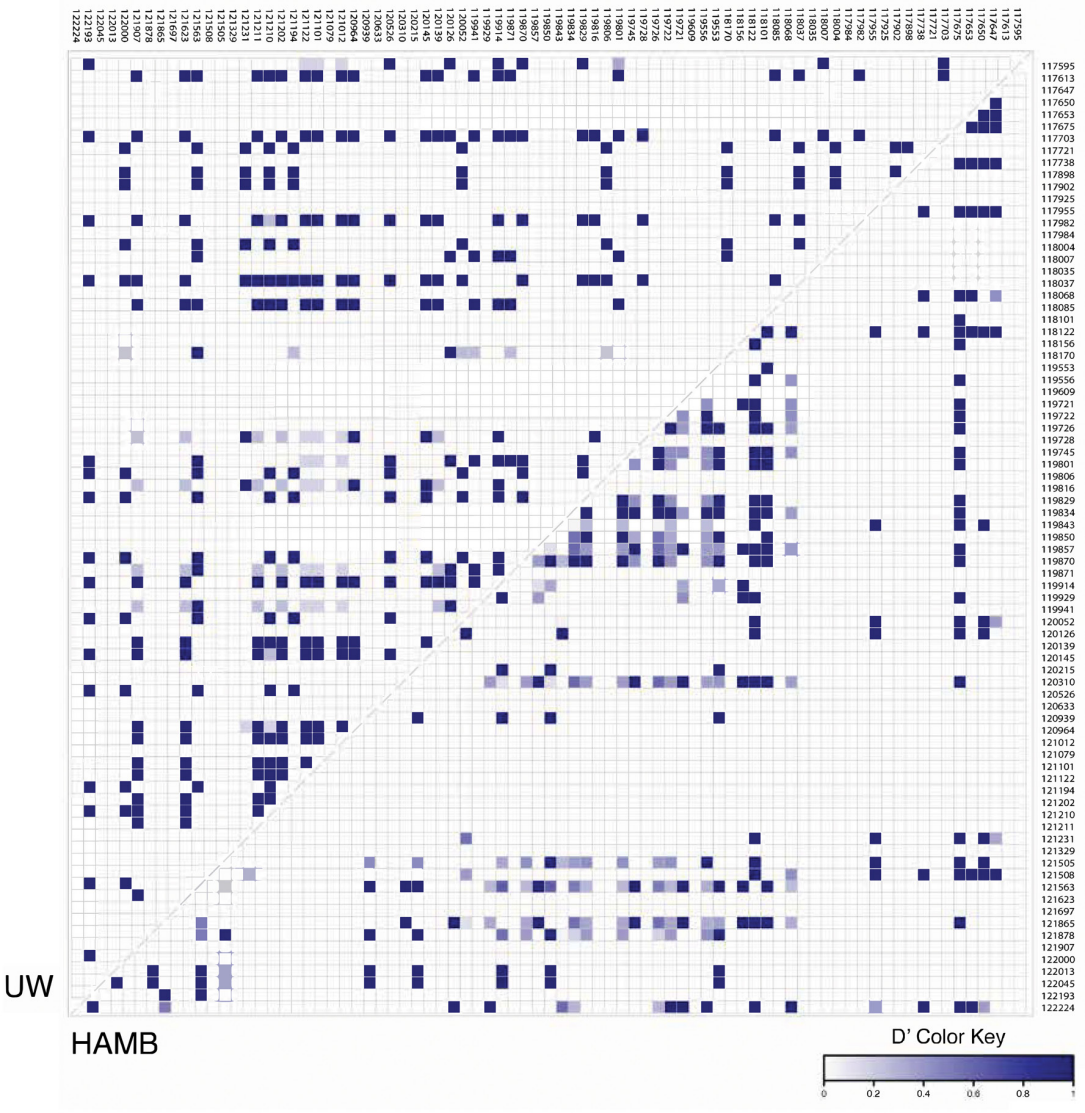
Pairwise linkage disequilibrium (LD) plots of UW and HAMB patients from the KSHV K12 and microRNA cluster region Localization of microRNAs genes is indicated on the top and the side, with nucleotide positions based on BCBL-1 genomic coordinates. A strong LD (D’) is indicated by a darker shade of blue (D’ close to 1) whereas white represents no LD (D’ = 0). The LD was calculated as Tajima's D’ with VCFtools 0.1.15. A total of 79 of the original 102 sites were kept after filtering.

In turn, while the microRNA cluster sequence was highly conserved, the pattern of SNPs in KSHV strains from South African and HAMB patients were distinct. Polymorphisms from each group were examined for linkage using VCFtools [17]. Analysis indicated that some SNPs were non-random and linked within groups, and the pattern differed between UW and HAMB as shown in Figure 3.

### microRNA Expression in biopsies

**Figure 4:**
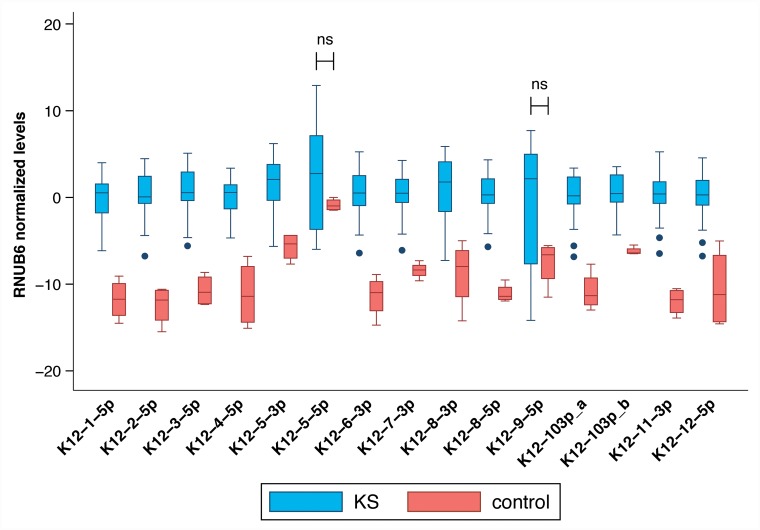
Comparison between mature KSHV microRNA expression in normal skin vs KS tumor biopsies Log transformed levels, normalized to the host transcript RNU6B are shown. All comparisons were statistically significant with p ≤ 0.004 except K12-5-5p^*^ and K12-9-5p which were not significant.

Mature KSHV microRNAs levels were measured using commercially available small RNA qRT-PCR assays and normalized in each sample to the levels of endogenous RNU6B; consistently higher KSHV microRNAs levels were detected in KS tumor biopsies compared to non-KS skin tissue as shown in Figure 4. Only microRNAs miR-K12-5-5p^*^ and K12-9-5p did not have significantly elevated levels in KS tissues. No association between mature microRNA levels and HIV status or ethnicity was observed.

**Figure 5:**
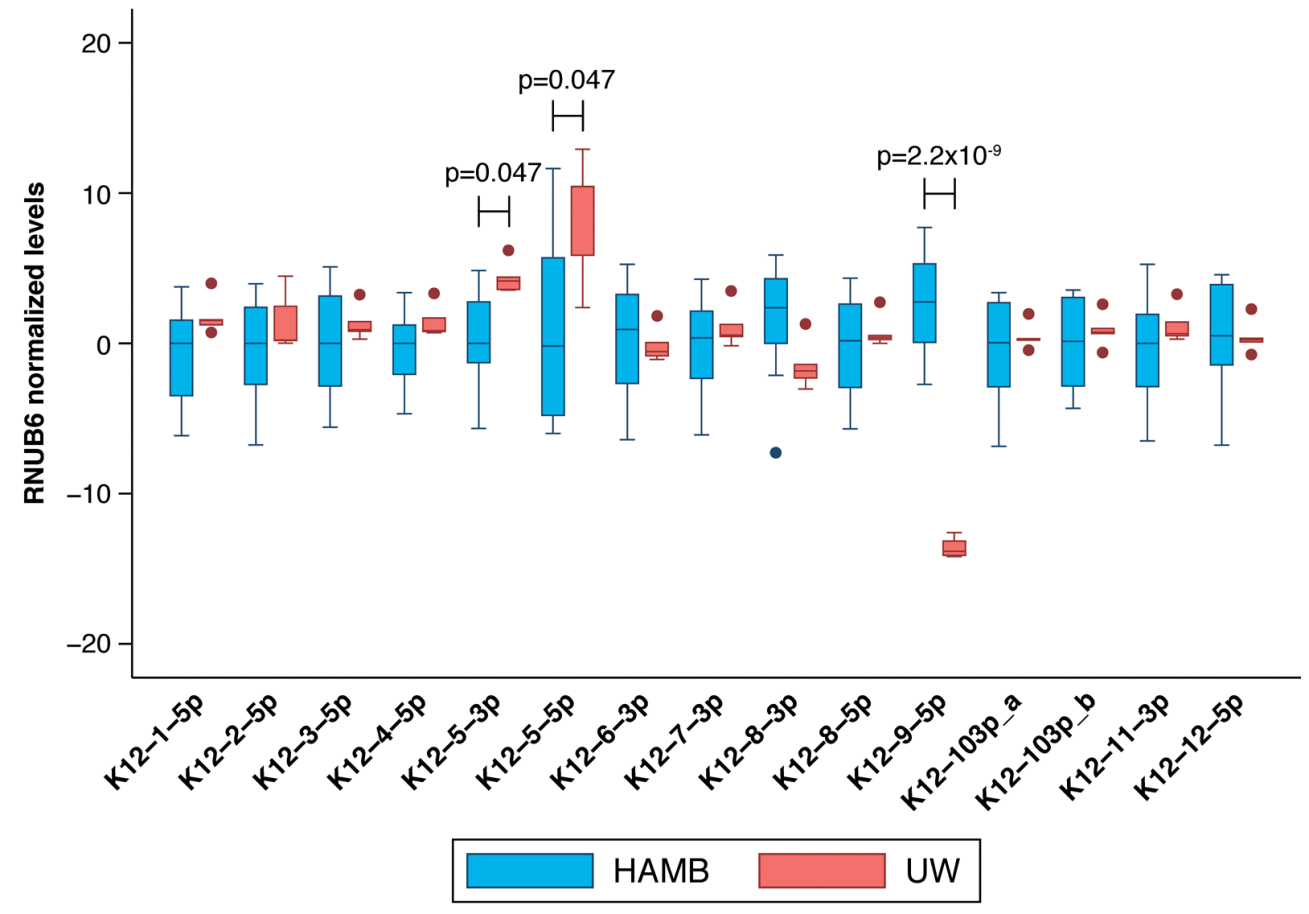
Comparison between mature KSHV microRNA expression in KS tumor biopsies from HAMB and from UW Log transformed levels, normalized to the host transcript RNU6B and to control samples are shown. Statistically significantly elevated microRNA expression was noted in K12-5-3p and K12-5-5p in samples from South Africa compared to the U.S., while K12-9-5p was decreased.

Although the estimated KSHV viral load was similar in UW and HAMB tumor tissues (median 5.7 log10 copies/10^6^ cells; IQR 4.6-5.9 vs. 5.6, IQR 5.5-6.6) many mature KSHV microRNAs were detected at higher levels in UW biopsies compared to HAMB except K12-8-3p and 9-5p, which were depressed (Figure 5). The same significant differences were observed when restricting the analysis to samples from HIV- infected individuals. While batch effects due to sample collection cannot be ruled out, we normalized all data using the endogenous RNU6B RNA which would be similarly affected. We observed a small measurement error (median coefficient of variation less than 20%) within individual replicates across all assays and both groups except for microRNA 9-5p in UW due to very high Ct values near the assay limit of detection.

### Correlation of microRNA SNPs and mature microRNA levels

When we examined the global effects of sequence variation on mature microRNA levels, we observed that SNPs detected in pre-miR K12-9-5p, 4-5p, 5-3p, and 7-3p were all associated with downregulation of K12-9-5p, which was detected at lower levels in samples from UW patients compared to HAMB (Figure 5). A highly-conserved SNP, located two nucleotides upstream from the start of pre-microRNA K12-3, was associated with downregulation of mature microRNAs K12-8-3p and 9-5p but an upregulation of K12-5-5p^*^ as summarized in Table 2.

**Table 2:** Summary of KSHV mature microrna expression differences associated with specific conserved single-nucleotide polymorphisms

SNP within	Nucleotide position (change)	Effect on	Regulation	Fold change	p-value^#^
K12-3	121617	K12-5-5p	up	69	6.64^*^10^-03^
		K12-6-3p	down	-51	5.98^*^10^-03^
	(A to G)	K12-8-3p	down	-80	7.02^*^10^-04^
		K12-9-5p	down	-1584	4.96^*^10^-05^
		K12-10-3p (a)	down	-27	1.36^*^10^-02^
		K12-10-3p (b)	down	-10	1.25^*^10^-02^
		K12-12-5p	down	-41	7.31^*^10^-03^
K12-4-5p	121449 (C to T)	K12-9-5p	down	-10164	6.98^*^10^-06^
K12-5-3p	121315 (A to G)	K12-5-5p	up	121	2.48^*^10^-02^
		K12-9-5p	down	-594	2.48^*^10^-02^
K12-7-3p	120386 (C to A)	K12-9-5p	down	-24354	6.33^*^10^-07^
K12-9-5p	119361 (C to A)	K12-8-3p	down	-73	1.27^*^10^-02^
		K12-9-5p	down	-21218	3.13^*^10^-07^

The nucleotide positions reference the BCBL-1 cell line (GenBank accession number AY973824.1). Many SNPs appear to influence the mature expression of not only the specific microRNA where it is located, but also the surrounding microRNAs. The small RNA assays for K12-10-3p (a) and (b) used a primer that only differed by one nucleotide and therefore cross-reactivity cannot be ruled out. # Benjamini-Hochberg corrected.

### *In silico* mature microRNA structure prediction

To ascertain the effect of the sequence variations identified on the structure of the KSHV microRNA cluster region, we determined, *in silico*, the secondary structure [18] of representative KSHV microRNA cluster sequences from a UW sample (UW5) and a HAMB patient (HAMB10) as shown in Supplementary Figure 1A-1B. In addition, for comparison we generated structural models for the BCBL-1 and VG-1 cells lines which share sequence similarities with HAMB10 and UW5, respectively (Supplementary Figure 2A-2B). Corresponding mature microRNAs expression raw Ct levels are shown in Supplementary Figure 3.

In sample HAMB10, the levels of all examined mature microRNAs within pre-miR-K12 cluster are lower than those observed in UW5 with the exceptions of miR-K12-8-3p and 9-5p, which are detected at higher levels. Also, miR-K12-5 contains an A→G SNP (nt121315) which closes the internal loop, predicted in BCBL-1, with an additional GC base pair. However, the remainder of the miR-K12-5 structure, as well as secondary structures surrounding that region, are identical to those observed for BCBL-1. The structure of the HAMB10 miR-K12-8 hairpin does not differ from that observed for BCBL-1. However, multiple SNPs flanking this region likely affect the conformation of nearby hairpins. Other microRNAs retain almost unchanged secondary structures regardless of SNPs. For example, in miR-K12-1, deletion of the C (nt 121685), involved in a G·C base pair in BCBL-1, was compensated by formation of a G·U wobble involving a previously unpaired U, thereby preserving the hairpin. The remaining SNPs in HAMB10 are located outside the pre-microRNA hairpins, frequently within sequences directly preceding the 5’ end of the pre-microRNAs, i.e., in miR-K12-3 A→G SNP (nt 121617). It has been previously shown that these regions are essential for DGCR8 recognition and Drosha cleavage [19, 20]. Thus, decreased microRNA levels in HAMB10 could reflect altered three-dimensional topology of the cluster that impairs binding of processing enzymes.

An interesting trend was observed in the UW5 sample where, although both pre-miR-K12-4 and pre-miR-K12-7 include single SNPs near their apical loops, their levels are comparable to those in BCBL-1 cells. Changes in mature microRNA levels were noted mainly for miR-K12-5-5p^*^ and 5-3p, miR-K12-9-5p and miR-K12-6-3p. MiR-K12-5-5p and 5-3p were detected at higher levels; the pre-miR-K12-5 region undergoes structural rearrangements arising from a A→G SNP and multiple flanking nucleotide changes. MiR-K12-9-5p and miR-K12-6-3p showed a decreased level. In pre-miR-K12-9, a C→A SNP was detected whereas in pre-miR-K12-6, we identified a G→A SNP which modifies a G·U base pair to A·U base pair, preserving the secondary structure of the region.

## DISCUSSION

We previously described KSHV microRNA sequence heterogeneity in PEL cell lines and clinical samples [14], and showed that certain SNPs in KSHV microRNAs were associated with MCD risk [15]. We then functionally analyzed the relationship between those SNPs, predicted microRNA secondary structure, microRNA production, maturation and targeting efficiencies using *in vitro* systems. We demonstrated that SNPs originally observed in KSHV-infected patients are associated with significant yet complex effects on microRNA maturation [16]. In this report, we further investigate SNPs in the entire microRNA region and illustrate *ex vivo* the association between sequence variation and changes in mature microRNA levels in KS lesions; we also predict the structure of pri-microRNA with wt and variant sequences.

Since our first report of KSHV microRNA sequence polymorphisms in 2010, the contribution of microRNA variations in the context of gene regulation and viral disease has increasing become more apparent as recently summarized [21]. Our current sequence analysis has identified new SNPs within the microRNA coding regions, many that are KSHV subtype specific, distinguishing viral strains commonly observed in Africa from Europe/North America. Sequence specific polymorphisms previously reported by us and others were verified including changes occurring in pre- and mature microRNAs. Most sequence variations observed occur in the pri-microRNA regions with a noted concentration of changes between viral mir-K12-8 and mir-K12-9 as detailed in Figure 2.

Analysis of viral mature microRNA expression revealed that all KSHV mature microRNAs examined were detected at elevated levels in KS tissues compared to control skin except for K12-9-5p and the star sequence K12-5-5p^*^. We included all 13 predominantly expressed mature KSHV microRNAs and two star sequences, identified in previous studies as important, in our current analysis [8, 14, 15]. Upregulation of KSHV microRNAs in KS has been previously reported [22]. Mature microRNA expression was observed in every KS biopsy in the current study. KSHV mature microRNA expression levels differed between the HAMB and UW groups, correlating with sequence differences between the two groups consistent with our previous *in vitro* observations [16]. Differences in mature microRNA levels in KS lesions from South Africa compared to US patients were not related to KSHV viral load or any other patient-related factor.

It has been suggested that dysregulation of microRNA production within lesions has implications in KS pathogenesis [23]. In this study, we observed that specific SNPs were associated with significant differences in the expression of the corresponding mature microRNAs, whether the SNP was localized within the stem loop, in the mature microRNA, or in the pre-microRNA. Moreover, the presence of certain SNPs correlated with changes in the level of mature microRNA expressed elsewhere in the cluster region or, in the case of pri-K12-3 SNPs, microRNAs expressed within the K12 locus (Table 2). The mir-K12-3 SNP was previously described by our laboratory as over-represented in sequences of African origin as well as predominant in cases of MCD and KICS [8, 15]. This SNP was functionally characterized by Manzano et al as a viral mimic of the human miR-23 family microRNAs. This polymorphism alters the seed region sequence potentially changing not only the recognized mRNA targets but also the specificity of binding to those targets [24].

It is unlikely that single polymorphisms are entirely responsible for RNA structure changes affecting the processing of adjacent microRNAs; instead, they may be associated with patterns of sequence variations, common to viral strains, that result in the changes observed as previously reported and shown in Supplementary Figures 1 and 2 [15]. Linkage distribution (LD) plots of the microRNA cluster region in UW and HAMB patients show distinct patterns of LD in each group supporting this hypothesis (Figure 3). We have previously observed linked SNPs that statistically significantly increased the probability of developing MCD [15].

Of note, all the sequence variations reported in Table 2 down-regulate the expression of mir-K12-9-5p. Umbach et al reported previously that a KSHV cell line, BC-3, did not express K12-9, presumably due to sequence variations [25]. We noted similar variations in the sequence of pre-K12-9-5p that are common in KSHV subtypes B and A5 [8, 15] and corroborated subsequent loss of expression noted by Umbach [16]. We now observe sequence variations in miR-K12-3, 5, and 7 that also appear to specifically target miR-K12-9-5p.

In this study, we report that the specific localization of SNPs within KSHV pri-microRNA cluster has a significant effect on levels of KSHV mature microRNAs. The observed effect is likely due to direct influence of SNPs on secondary structure and tertiary contacts within the microRNA cluster, which in turn affect the accessibility of processing enzymes to their target RNA binding sites [26, 27]. MicroRNAs that reside on clustered or polycistronic transcripts represent a unique regulatory system, where individual “units” can be processed with diverse efficiencies despite being co-transcribed [28–30].

The presence of SNPs also regulates microRNA biogenesis. Both the 5’ end of microRNA that is generated from the 5’ arm of the pre-microRNA (5p) by Drosha, and the 5’ end of microRNA that is produced by Dicer from the 3’ arm of the pre-microRNA (3p), are under selective pressure to be highly conserved. The sequence preceding the 5’ end or trailing the 3’ end of the pre-microRNAs, forms an imperfect stem that is recognized by DGCR8 as part of the required structure for Drosha cleavage [19, 20]. The terminal loop is also important for Dicer/TRBP complex binding [31], as well as for other protein binding [32]. Thus, a single nucleotide change within any of these sequences can shift the processing sites during microRNA biogenesis, which could result in microRNAs with alternative target-spectra and/or disrupt microRNAs production.

The importance of sequences flanking the pre-microRNA in mature biogenesis has also been reported in both human and viral miRNA; SNPs located within pre-microRNAs have also been shown to affect the Drosha/DGCR8 processing step. In particular, this was demonstrated to occur with SNPs in KSHV miR-K12-5 [33, 34]. These results suggest that the sequence surrounding microRNA and unknown factors with which it interacts might affect its folding or protect its structural conformation against changes caused by some mutations. In our data, the effect of sequence variations was mostly noted in the pri-microRNA regions of K12-5, -8, and -9, resulting in significantly different levels of mature microRNA.

Our BCBL-1 secondary structure prediction is similar, yet not identical to that obtained for pri-miR-K10/12 transcript by SHAPE [35]. This result can be assigned to the different sequence length and prediction methods applied. Furthermore, SHAPE analysis requires that the RNA sample assumes a uniform conformation in solution, which is difficult to achieve for long transcripts, such as pri-miR-K10/12. If conformers are not separated and probed by in-gel SHAPE [36], the structural models will represent averaged structures comprising contributions from all conformers in the respective mixtures. Here, and as shown previously [28, 29], the practicality of *in silico* secondary structure predictions for microRNA cluster, can resolve that issue.

Secondary structures predictions cannot elucidate tertiary structure and accessory protein interactions that may affect the stability of hairpins contributing to processing efficiency of microRNA transcripts. Moreover, we did not measure microRNA levels for all star sequences, including K12-9-3p, which may be informative. The specific action of the newly identified variations in this study on mature microRNA processing, whether the binding of Drosha or Dicer is directly affected, was not determined and requires further elucidation. However, we previously demonstrated changes in Drosha/DGCR8 and or Dicer processing associated with KSHV strain specific variations within pre-microRNA sequences miR-K2, 5, 6, 7, 9, and 10. These variations are highly conserved, occur within samples in the current study, and can influence the mature expression of surrounding microRNAs as shown in Table 2 [16].

In conclusion, although the structural characteristics and sequence elements required for microRNA processing are incompletely defined, our data, in the context of the current literature, suggest that microRNA processing can be profoundly altered by SNPs in the pri-, pre-, and mature microRNA sequences. This is the first study to examine KSHV mature microRNA levels in tumors from diverse patients infected with different virus strains, representing all major viral subtypes, except for the rare D and E subtypes. While further studies are needed, our results suggest that KSHV sequence variation that occurs within African A5/B/F subtypes influence the expression levels of mature microRNAs, potentially changing the specificity for mRNA targets, which can lead to altered regulation of viral gene products and key cellular pathways, modifying KS pathogenesis.

## MATERIALS AND METHODS

### Patients and samples

Patients were recruited from the NCI HIV and AIDS Malignancy Branch Clinic, Bethesda MD, USA (HAMB) as well as the Dermatology clinic at the Charlotte Maxexe Johannesburg Academic Hospital and the University of Witwatersrand, Johannesburg, South Africa (UW). Patient characteristics are shown in Table 1. All UW patients were African men with AIDS-KS, whereas HAMB patients were individuals of both sexes including a variety of ethnic background and geographical provenance, with or without HIV infection. Punch biopsies were obtained from 24 patients with suspected KS: pathologic examination of biopsies confirmed KS in 19, and the remaining 4 were used as controls (Table 1). Three control biopsies from the HAMB were obtained from patients with a previous history of KS. KS history was not available for control UW1. All KS tumor tissues were obtained from patients prior to treatment. Biopsies from South African patients were stored in RNAlater and shipped to the Frederick National Laboratory, while samples from HAMB were collected in TRIzol. HAMB and UW patients were enrolled in one or more clinical studies evaluating KSHV associated diseases (NCT01495598; NCT00006518 and M090515 respectively). The studies were approved by the relevant IRBs. All participants provided written informed consent in accordance to the Declaration of Helsinki.

### Specimen processing

Punch biopsies were homogenized in Trizol reagent (Thermo Scientific) using a gentleMACS dissociator (Miltenyi Biotec). Total RNA was first extracted using a modified protocol from RNAzol (Zymo) according to the manufacturer's instructions for samples collected in Trizol. Afterwards, DNA was back-extracted following the Trizol reagent protocol (Thermo Scientific). Both RNA and DNA were quantified using a Nanodrop 1000 (Thermo Scientific).

### KSHV viral load

KSHV viral load in biopsies was measured using a quantitative real-time PCR assay as previously reported [37]. A cell quantitation assay based on the ERV-3 gene [38] was used to calculate viral copy numbers per cell. Assays were performed in triplicate using approximately 250 ng total DNA per reaction.

### Amplification, cloning, and sequencing of KSHV K1 and microRNA genes

The K1, T0.7, and microRNA cluster regions were amplified via nested PCR and sequenced as described elsewhere [8, 14, 15]. Cloning of amplified products was performed using pGEM-T Easy vector system (Promega). Viral subtype in controls with low or non-detectable viral load in skin samples was determined using corresponding saliva or lymphocyte DNA, where available. Overlapping forward and reverse sequencing reactions were performed for each region/fragment sequenced, and the sequence reads were collected using an ABI Prism 3100xl Genetic Analyzer (Applied Biosystems). A minimum of two clones were sequenced for each gene region.

### Phylogenetic analysis

All sequences were assembled using DNA Baser and identities were confirmed using BLAST (v2.91.5). Nucleic acid sequences were used for the T0.7 and microRNA cluster regions but K1 gene sequences were translated to amino acid prior to alignment in Clustal X (2.0). Appropriate reference sequences were obtained from GenBank and previous studies; all sequences were trimmed to equal lengths in Genedoc (v2.7). Phylogenetic trees were constructed using Bootstrap neighbor-joining analysis generated in MEGA v6.

### Quantification of mature KSHV microRNAs

Mature KSHV microRNA levels were measured using custom TaqMan small RNA stem loop real-time qRT-PCR assays (Applied Biosystems) as previously described [16]. Sequence specific assays were used for each sample based on the sequence of the respective mature microRNA. The human small nuclear RNA RNU6B was used as the endogenous control and for normalization. The 2^(-ddCt) method was used to calculate the fold changes referencing both the human small nuclear RNA RNU6B and normal skin tissue as the experimental and control conditions. All samples were tested multiple times in triplicate reactions; Ct values of the replicates were averaged from a minimum of 6 independent reactions. Negative control wells were included for each assay in triplicate. Ct values above 38 in both sample or control wells were considered non-specific amplification. All qRT-PCR reactions were performed using an ABI Prism 7900HT sequence detector system.

### RNA secondary structure prediction

RNA secondary structures for pri-microRNA clusters sequences were generated using the RNAstructure software package (ver. 5.7) [39]. The final structures were generated in VARNA (v.3-9) [40] using NAView drawing algorithm [41]. The models were edited to account for structural intersections between tight folds, and the position of SNPs was color annotated.

### Statistical analyses

Target genes (microRNAs) levels were normalized to endogenous RNU6B. Comparisons in DNA VL and microRNA levels across categories were performed with moderated *T* tests or nonparametric test, according to the dependent variable's distribution. Statistical analyses were performed using GeneSpring v14.8 (Agilent Technologies) and Stata v13 (StataCorp). For the heat map shown in Figure 3, linkage disequilibrium was calculated as Tajima's D’ with VCFtools 0.1.15 [17]. Of the 102 sites included, 79 sites were kept after filtering.

## SUPPLEMENTARY MATERIALS FIGURES



## Abbreviations

KSHV: Kaposi's sarcoma-associated herpesvirus
MCD: multicentric Castleman's disease
KS: Kaposi's sarcoma
SNP: single nucleotide polymorphism
PEL: primary effusion lymphoma
KICS: KSHV inflammatory cytokine syndrome
wt: wild type
HAMB: HIV and AIDS Malignancy Branch
UW: University of Witwatersrand
nt: nucleotide
