# Supplementary material for: Polymorphisms in KSHV-encoded microRNA sequences affect levels of mature viral microRNA in Kaposi Sarcoma lesions
Source: Oncotarget. 2018 Nov 9;9(88):35856–69. doi: 10.18632/oncotarget.26321 (PMC6254685; doi:10.18632/oncotarget.26321)
Supplement: Supplementary file 1 [file oncotarget-09-35856-s001.pdf]

Polymorphisms in KSHV-encoded microRNA sequences affect levels of mature viral microRNA in Kaposi Sarcoma lesions

SUPPLEMENTARY MATERIALS

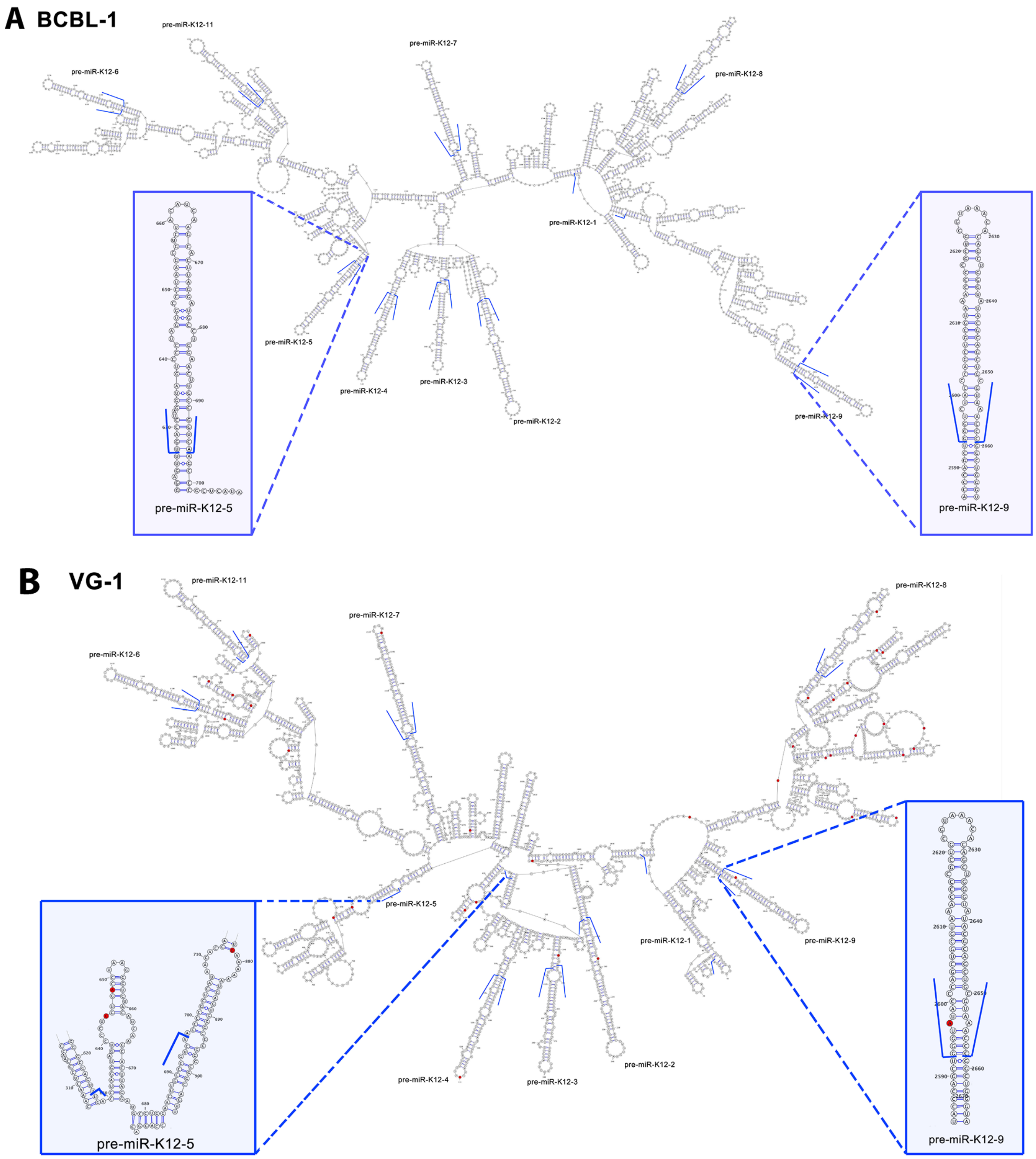

Supplementary Figure 1: (A and B) *In silico* RNA secondary structure prediction is shown for the microRNA cluster region of the BCBL-1 cell line, used to represent the reference sequence, and the more variant VG-1 that encodes many of the SNPs identified in clinical samples (indicated in red). Examples of structure variations within the pri- and pre-microRNA regions are illustrated for K12-5 and K12-9. The individual pre-microRNAs are labeled, and marked with blue bracket lines, the SNP are indicated in red. BCBL-1 GenBank accession number AY973824.1.

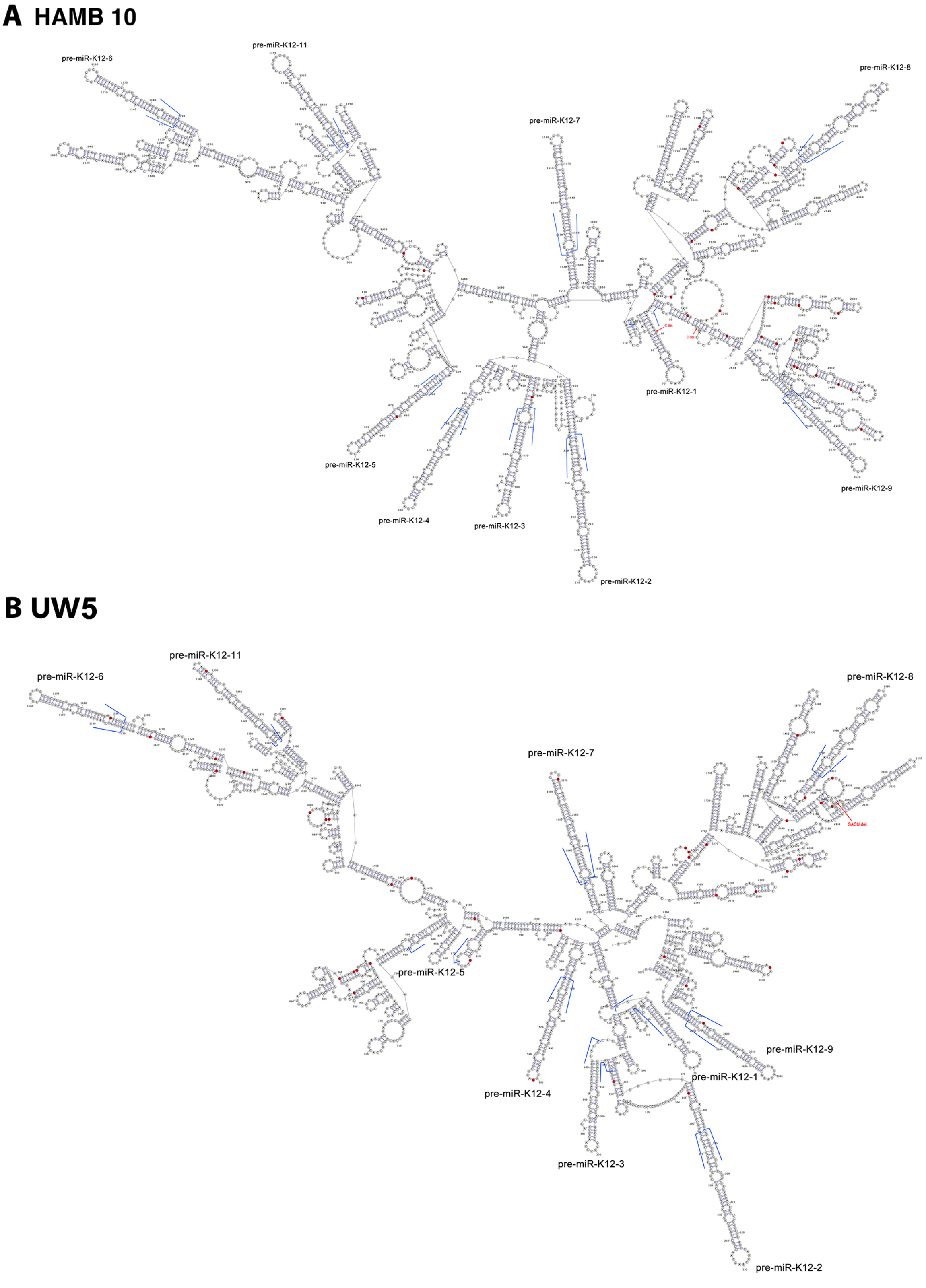

Supplementary Figure 2: (A and B) *In silico* RNA secondary structure prediction is shown for the microRNA cluster region of the UW5 and HAMB10 cell lines. The individual pre-microRNAs are labeled, and marked with blue bracket lines, the SNPs identified in clinical samples are indicated in red. The sequence of HAMB 10 closely resembles that of the BCBL-1 cell line and is used as a representative of KSHV viral strains from North America/Europe. The UW5 sample has SNPs found in the VG-1 cell line and is a representative of African KSHV strains. The specific SNPs for each are detailed in Figure 2.

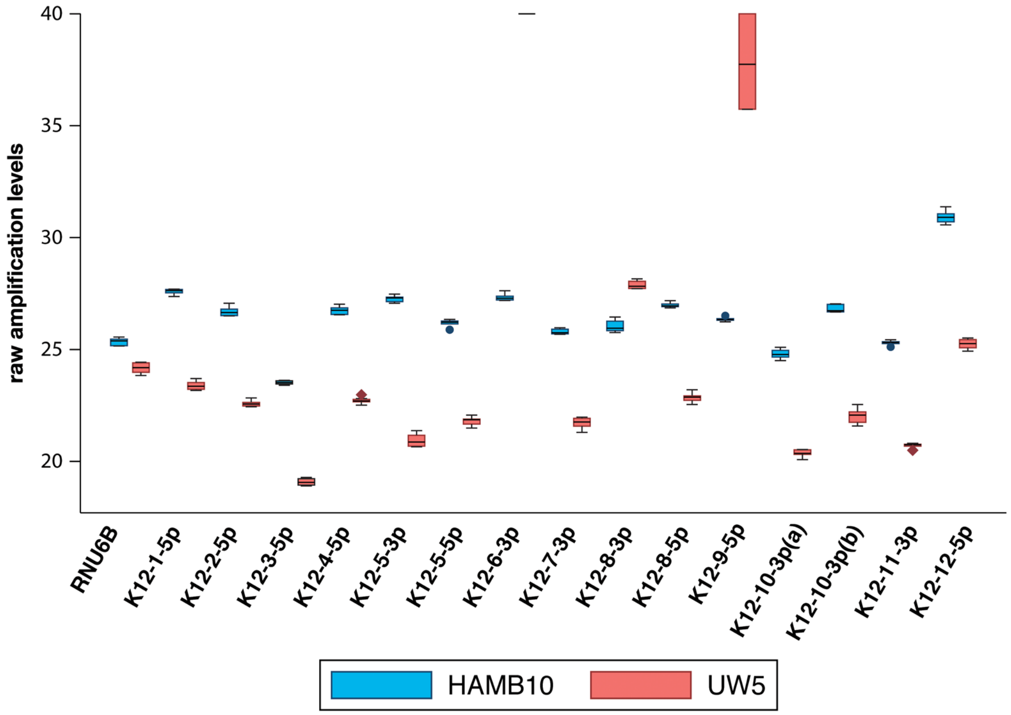

Supplementary Figure 3: Comparison between mature KSHV microRNA expression in the HAMB10 vs the UW5 KS tumor biopsy. Raw amplification [C(T)] values are shown. The distribution is calculated using a minimum of six technical replicates. Measurement error was small (median coefficient of variation was less than 20% across all assays and both groups, except for microRNA 9-5p in UW, due to very high Ct values near the limit of detection of the assay).
